# The Action of Obstetrical Forceps**Archives de Tocologie de Maladies des Femmes et des Enfants Nouveau-nes*. March, 1876.

**Published:** 1876-07

**Authors:** 


### Translations.

### THE ACTION OF OBSTETRICAL FORCEPS.*

*(From the French of Prof. E. Hubert.)*

BY FRED. J. HUSE, M.D.

Few surgical instruments are called into requisition so frequently as the forceps ; few are so imperfectly understood in regard to method of action. The *iron hands* of J. Palfyn have been subjected to innumerable modifications in their mode of union, in length and form of handle, in amount of concavity, and shape of tip. Every obstetrician has been eager to effect an alteration by some addition or change, but the greater proportion of these modifications have been made at hazard, and more frequently as experiments than as the expressions of an established theory. "When one fails to understand the use of old forceps," said Velpeau, "he invents new." As the result, though progress may be trifling, instrument makers thrive.

In order to throw light upon the manner in which forceps may be brought to a condition of perfection, it is pre-eminently essential that the effort should be made with a full understanding of what may reasonably be expected of them. When this point has been definitely determined, science will have taken a long step in advance ; the purpose once indicated, its mode of fulfillment is easily established, and the completion of the instrument may be left to the artisan. But the disagreement which exists among the schools shows that this step has never been taken. Contrary purposes are still proposed, and what some consider progress seems to others recoil. This results from the notions, that to produce general satisfaction a single instrument ought to fulfill,

* *Archives de Tocologie des Mal idies des Femmes et des Enfants Nouveau-nes.* March, 1876.

equally happily, indications which are diametrically opposed; and at one time grasp the head without compressing it, at another compress it, in order to reduce it, in the act of grasping. These conditions can hardly be combined without much difficulty, and we do not propose to tarry in such a search, preferring rather to adopt the use of two forceps of different type, a *tractive* forceps and a *compressive* forceps, each adapted to conditions and requirements entirely distinct.

In 1860, my father carefully investigated the method of using forceps as a means of traction.* For myself, I propose to sum up his teachings in a few words, and then, placing myself upon another stand-point, examine the instrument as a means of reduction.

I.

The following propositions were demonstrated by my father by calculus:

1st. Tractions made in the direction of the axis of the canal are entirely utilized.

2d. Tractions perpendicular to this axis are entirely expended in producing compression.

3d. Oblique tractions are resolved into extractive, useful force, and compressive or injurious force. The useful force is diminished and the injurious force increased in proportion as the direction of traction is removed from the centre of the head; as the head deviates from the pelvic axis; and according as the forceps are grasped further from the joint.

Though excellent in the inferior strait, where the traction can be directly opposed to the resistance, the forceps become of less value at the superior strait, because their action is no longer *in* the direction of the axis, being, at most, only *parallel* to it. As a boat drawn by a tow-line is immediately dragged against the bank unless the rudder, so turned as to produce a strong resistance on the part of the water, serves to maintain its axis in the axis of the canal; so in the pelvis the anterior wall may be compared to the rudder, in presenting that resistance which maintains the head in the central line of the resistance of the pelvis, despite the defect of traction.

Now, as we have already indicated, this resistance may be resolved into compression and the neutralization of force. In order to avoid bruising of tissue and loss of

* Memoires de l'Académie Royale de Med. de Belgique, tome IV, 1860.

force, my father caused forceps to be made in the form of the letter S, thus rendering possible the most exact prolongation of the direction of strain in the axis of the superior strait. A more simple means of accomplishing the same result consists in perforating the handles of ordinary forceps with two holes near the joint, and adjusting an iron stock to the instrument by means of a couple of fids.

Still another method of giving the most serviceable direction to tractions consists in rendering the handles of the instrument stable with one hand and applying the force at the joint ; thus it is changed into a powerful lever, and its blades are caused to describe a curve which, like the axis of the strait, has a downward and backward direction. There is, however, this single objection to this mode of use, that if the head has not been seized with care, the blades may slip off suddenly and wound the posterior wall of the neck, vagina, or perineum.

### II.

It is the general opinion of the present day, that the classical forceps is a poor instrument of reduction, and every-day practice demonstrates that it can hardly reduce the head many millimetres without greatly compromising the existence of the fœtus. An infant, which, in order to be born, must submit to a diminution of a centimetre in one of its cranial diameters, can readily be brought forth alive by the exertions of the organism ; but it is almost surely lost if subjected to the forceps.

Art is, therefore, still far behind nature, and it is interesting to examine how it is that such different results are brought to pass.

Nature expels the head by leaving it at full liberty to turn toward the largest amount of space ; the compression is slow, graduated, and the reduction is never carried beyond the point strictly necessary ; the head is only compressed between two points, and while one diameter yields under the strain, all the others can easily expand. In other words, the *cranium is changed in form, without notable change in capacity.*

The Flemish lever,* in skillful hands, may very nearly imitate the natural mechanism, but it is not so with ordinary forceps. The head grasped in the direction of its length by the ellipse of the instrument, compressed

* Of the third class.

transversely by the blades and backward by the bones of the pelvis, can scarcely expand except in its vertical diameter; but this expansion does not sufficiently counteract a reduction, which is effected in all other directions by crushing. Alteration of form on one side, alteration of capacity on the other, such difference in the mode of action is sufficient to account for the difference in result.

From another point of view, the cephalic curvature, which allows the instrument to adapt itself to the convexity of the cranium, prevents compression at the desired locality. After a head has been laboriously extracted, the principal marks impressed upon the integument are found upon the face, where the effect has been exerted upon an irreducible diameter, while, at the same time, the parietal eminences have almost entirely escaped the action of the blades.

It is also essential to bear in mind that the obstacle at the superior strait nearly always consists in a shortening of the antero-posterior diameter, and that it is impossible to place the instrument there.

No doubt the head diminishes in volume under the force of energetic traction, just as iron is drawn out in the rolling mill, but at the expense of the resistance of the bony circle, across which it is dragged, and in direct opposition to the defective pressure of the instrument; the proof of this being that reduction is much more easily accomplished if, instead of binding the cranium with the steel hoops of the forceps, traction is made by means of an elevator fastened in the vertex; or, indeed, as Simpson has proposed, by means of a disk of leather closely adapted to the head by means of an air-pump.

It would, therefore, be advisable to renounce the forceps as an instrument of reduction, unless such modifications can be effected as shall remove, at least in part, these glaring defects. The process of determining rational modifications is very complex, since the following problems present themselves: How much reduction of the head is compatible with the preservation of the infant's life? What force can be exerted upon the mother and infant without too much risk? What form and what size shall be given to the instrument, in order to render it the most efficient and the least destructive?

*Form of instrument.* The best forceps is evidently that which in action will most closely imitate nature and diminish the volume of the head in the most harmless manner. "However defective may be the contrivance of a forceps," M. Chassagny has said, "it will never be absolutely bad if the blades are long; but with short blades, the most skillful man will only be able to construct a forceps which will be detestable in every respect." He then demonstrated, by calculus and by most ingenious experiments, that the longer the blades of a forceps are, from the joint to the tips, the better they adapt themselves to the convexity of the head; the less prominent their cephalic curvature, the less oblique their compression, and consequently the less they are obliged to compress the head in order to retain it. On the contrary, the more the blades are shortened, the more they must separate in order to include the head; the more downward must be their curvature, in order to fit upon it; the more firmly must they be closed, to prevent slipping off, and the more they affect by their bite. Moreover, when held between parallel blades, the head can be more easily elongated than between crossing blades.

The genius of Palfyn, therefore, in 1721, gave a form to the handles of forceps from which it was unwise to deviate, and which reason now restores. To cross the blades was to shorten them, and this was to spoil them.

*Reductibility of the fœtal head.* When a head is compressed with strong crossed forceps, it may be seen to be slightly flattened and elongated. The bones overlap and the scalp is wrinkled. If pressure is increased, the elongation becomes less and less apparent, and, strange to say, the overlapping disappears and the skin is put on the stretch. At first the brain, being thus forcibly compressed, resists in the manner of a fluid, and reacts in all directions. Under further increase of pressure, either the forceps bend, or the bones are broken, or the brain, reduced to pulp, escapes through the orbits, nares, sutures, and the occipital foramen. In this manner the head is rendered sufficiently ductile to allow of its passing through an opening of five centimetres, but evidently this is not the desired result; a reduction is desired which is less brutal, and which is compatible with the life of the infant.

Beaudelocque made observations which are still famous upon nine fœtal heads. Seized in selected forceps, these were reduced in different degrees, but always more than a centimetre, and the diameter perpendicular to the diameter of seizure was with difficulty elongated a few millimetres. Pétrequin, experimenting in the same manner, arrived at quite different results, and ascertained a constant elongation proportional to the cranial diameter opposed to the diameter under compression.

The source of these differences, according to M. Chassagny, may invariably be found in the form and temper of the forceps employed. Had they been in agreement, however, these observations would still have failed to possess any considerable value in our estimation, for they were made in the laboratory, and under conditions which do not present themselves in nature. In order to become instructive and convincing, they should be commenced afresh within artificial pelves, which may register the compression of force applied regularly and uniformly, and by means of forceps of various sizes, shapes and tempers. Such were the experiments of M. Chassagny, and he arrived at the following results:

1. The same head having been seized by different forceps in the direction of its biparietal diameter, the reduction of this diameter was zero with the forceps of M. Pajot, five millimetres with the forceps of Hatin, and one millimetre with the forceps of M. Chassagny.

2. To obtain these varied degrees of reduction, the pressure of crossed blades was quadruple that which was used upon the forceps of M. Chassagny.

3. When the head was seized from a frontal eminence to the opposite occipital eminence, the greatest separation of sinus was 101 millimetres with the forceps of M. Chassagny, 115 millimetres with that of Hatin, and 130 millimetres with that of M. Pajot.

4. To draw the same head through the same strait, when it required a force of 18 kilogrammes with the forceps of M. Chassagny, it required 25 with the forceps of Hatin, and 30 with that of M. Pajot.

5. In artificial pelves with movable walls, where the forceps of M. Chassagny did not produce any separation, that of Hatin caused one of five millimetres, and that of M. Pajot one of eight millimetres.

6. During traction the lips of blades which were not crossed did not approach each other, while those of the forceps of Hatin approached six, and those of M. Pajot seven, millimetres.

7. Not only was the diameter of seizure reduced more, the force exerted and the compression sustained by the pelvis less, under the use of the forceps of M. Chassagny than with the two others, but also the brain of the infant would have sustained much less injury, since an artificial head* filled with water, seized in turn by each of the three forceps, and drawn with equal force through the same pelvis, lost, during extraction, seven grammes of fluid with the forceps of M. Chassagny, thirteen with that of M. Hatin, and twenty with that of M. Pajot.

It is generally conceded that the vault of the cranium may yield, under certain conditions, and become level with the base ; so, although a reduction of about† fifteen millimetres in the biparietal diameter may be dangerous, it is not, therefore necessarily fatal, and it is conceivable that an infant could be born alive from a pelvis of seventy-five millimetres. Yet we are not of the opinion that because an infant could be *expelled* it could likewise be *extracted* alive, for it is a matter of observation that the forceps can hardly reduce the cranium, even a few millimetres, without grave hazard to the existence of the fœtus, while nature may accomplish much greater reduction with less danger. We have, indeed, seen several women with pelvic measurement of seventy-five millimetres expel living children, while in previous labors the forceps had invariably brought forth their children stillborn. To attempt to pull an infant at term through such a constriction was to expose it to almost certain death.

Perhaps, however, it may be allowable to hope that sustained mechanical traction, being less energetic and less brutal than manual labor, may yield more favorable results, and as it presents less gravity than all the other resources of art, the obstetrician is authorized in using it whenever insufficiency of uterine force is manifest.

* This head was of vulcanized rubber, solid in the portions corresponding to the base of the skull and the face, having to correspond with the cranial cavity; a vacant space was left, which could be filled with water. An opening allowed the water to escape during compression, and the quantity of water escaping afforded an exact measure of the diminution of capacity.

† We have used the expression *about* fifteen millimetres, because the biparietal diameter is never directly engaged in the strait, and it is in reality an oblique line near this diameter, but a trifle shorter.

(To be continued.)

